# False Protection of Real-Time Traffic with Quieting in Heterogeneous Wi-Fi 7 Networks: An Experimental Study

**DOI:** 10.3390/s23218927

**Published:** 2023-11-02

**Authors:** Andrey Barannikov, Ilya Levitsky, Evgeny Khorov

**Affiliations:** Institute for Information Transmission Problems of the Russian Academy of Sciences, 127051 Moscow, Russia; barannikov@wireless.iitp.ru (A.B.); levitsky@wireless.iitp.ru (I.L.)

**Keywords:** real-time traffic, 802.11be, Wi-Fi 7, channel access, R-TWT, quieting, quiet interval

## Abstract

To provide limited delays for remote sensing and control, gaming, and virtual reality applications, the Wi-Fi 7 standard introduces the Restricted Target Wake Time (R-TWT) mechanism, which reserves time intervals for particular stations with such real-time traffic. As legacy stations do not support R-TWT, the access point forbids channel access during these intervals for legacy stations. Quiet Intervals have been announced for this purpose. Since the support for the Quieting Framework can be configured as mandatory in some networks, Quiet Intervals are assumed to be valid protection for R-TWT. The paper describes experimental results with mass-market devices that disprove this assumption. The paper reveals significant inconsistencies between the standard and widely used devices, e.g., the inability to schedule multiple Quiet Intervals. It will be a significant problem for Wi-Fi 7 devices using R-TWT in heterogeneous networks with legacy devices and will require much effort from academia and industry to solve.

## 1. Introduction

With the development of wireless communications, Wi-Fi technologies have found more and more applications in different spheres of human activity. Recently, Wi-Fi developers have changed the paradigm from improving nominal data rates to providing guaranteed latency, throughput, and reliability. These guarantees are crucial for emerging real-time applications, such as remote sensing and automation, virtual reality, and mobile gaming, which require delays of the order of 1–10 ms and packet loss rates of about $10^{-4}\ldots 10^{-6}$ [1,2]. Providing such guarantees in wireless local access networks presents a challenge. It motivates researchers to test the feasibility of real-time data delivery in Wi-Fi both theoretically [2] and experimentally [3].

The satisfaction of the requirements of real-time applications in Wi-Fi networks is complicated because of the use of enhanced distributed channel access (EDCA). This mechanism is based on random channel access, where stations compete with each other to use the channel. It leads to significant delays and decreases in reliability due to collisions. Even though EDCA can reduce latency for voice and video traffic [4,5], it still cannot meet the demands of servicing real-time traffic. Moreover, the effectiveness of the EDCA mechanism highly depends on the number of devices [6].

To satisfy these requirements, IEEE 802.11be, also known as Wi-Fi 7—which is currently under development—defines a Restricted Target Wake Time (R-TWT) mechanism. It allows an access point (AP) and a station to negotiate a sequence of service periods (SPs) when the station is active and exchanges information with the AP. In contrast, the other Wi-Fi 7 stations are prohibited from transmitting. Although support for the R-TWT mechanism will become available in new commercial devices, a typical Wi-Fi network is heterogeneous: it contains devices of various generations. Thus, a Wi-Fi 7 network may contain legacy devices that do not support R-TWT and do not know about reserved SPs. To forbid channel access by legacy devices, the current version of the IEEE 802.11be standard [7] prescribes the usage of the Quieting Framework introduced in the old IEEE 802.11h standard [8], thus being backward compatible. With this framework, the AP can announce a series of Quiet Intervals, during which the stations are prohibited from transmitting frames. This framework has been introduced to allow the devices to measure the channel quality and to provide coexistence with other technologies.

Although the support for the Quieting Framework is optional, the AP may declare it mandatory in the network. So, in theory, the Quieting Framework is assumed to protect transmissions in reserved R-TWT SPs. In this paper, we disprove this assumption. We designed an experimental setup that was used to examine in detail how the parameters of the Quieting Framework influence the behavior of devices. We demonstrate significant inconsistencies between standard and specific implementations, which can hinder real-time applications in Wi-Fi 7 devices using R-TWT.

The paper has the following structure. Section 2 describes the Quieting Framework and reviews related works. In Section 3, we describe the experimental setup. Section 4 presents and discusses numerical results. Section 5 concludes the paper.

## 2. Quieting Framework and Related Works

**Figure 1:**
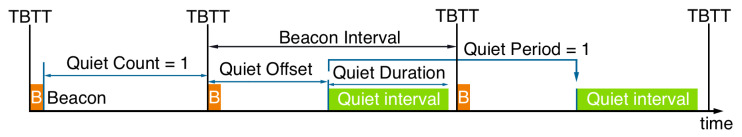
Announcement of Quiet Intervals.

IEEE 802.11h introduces the Quieting Framework as a part of the Dynamic Frequency Selection (DFS) framework [8,9]. This framework is used to detect signals from other devices, such as radars, that could interfere with the channel. During the Quiet Interval, stations remain silent, i.e., they are prohibited from sending any frames to allow the measurement of the channel. The schedule for the Quiet Interval is communicated through Quiet Elements, as shown in Figure 1. These Quiet Elements may be included in beacons, which are periodically broadcast by the AP. Each Quiet Element includes:

- Quiet Count, i.e., the number of beacons before the Quiet Interval;
- Quiet Period, i.e., the number of beacons between Quiet Intervals;
- Quiet Duration, i.e., the duration of each Quiet Interval expressed in time units (TU) of 1024 μs;
- Quiet Offset, i.e., the shift of the Quiet Interval from the target beacon transmission time (TBTT), measured in TU.

To make the Quieting Framework mandatory in the network, the AP enables DFS by raising the Spectrum Management (SM) flag in beacons and other suitable frames [10]. If a client device not supporting this feature wants to associate with the AP, the AP can reject its request, accepting only requests from SM-capable clients.

IEEE 802.11be prescribes protecting R-TWT with Quiet Intervals with a fixed duration of 1 TU. Multiple Quiet Elements can be used to protect different R-TWTs, e.g., for quasi-periodic virtual reality traffic.

Several papers have investigated various aspects of the Quieting Framework, focusing mainly on possible attacks on the network. In paper [11], the authors examined numerous combinations of Wi-Fi chips and operating systems in search of possible attacks on the Wi-Fi network using information forging in beacons. Only two out of six combinations implemented the Quieting Framework according to the standard. The majority of tested devices have problems with the Quieting Framework implementation; moreover, Windows devices do not support the Quieting Framework. The authors show that real devices have issues with the Quieting Framework implementation, which is related to the used operating system. This work reveals plenty of useful information about the Quieting Framework, but software has changed significantly since then, so the behavior of modern devices could be completely different.

The paper [12] studies some attacks that substitute some information in Quiet Elements. As the authors used invalid Quieting Framework parameters, their results do not apply to the case where Quiet Intervals are valid and protect R-TWTs.

In addition to security testing, the Quieting Framework is used to improve the performance of other mechanisms. The papers [13,14] investigate the Quieting Framework as a tool for contention reduction, prioritizing, or distributing traffic. In [13] the authors are focused on the AP’s ability to adaptively form Quiet Intervals for synchronizing the transmission periods of different STAs, taking into consideration the interference in the channel. However, the paper does not consider short Quiet Intervals of 1 TU for R-TWT and the support for several Quiet Intervals by devices.

Works [15,16] consider the Quiet Time Period mechanism, which is conceptually similar but serves a different purpose. It helps an AP to reserve channel time for STA-to-STA transmissions. The Quiet Time Period was introduced in the 802.11ax amendment and is supported only by Wi-Fi 6 or later devices. Like R-TWT, it also suffers from the presence of legacy devices and is unable to protect R-TWT SPs.

A similar issue is observed in the Restricted Access Window (RAW) mechanism, introduced in the 802.11ah amendment and studied in [17,18,19,20,21]. The RAW creates time intervals during which only a predefined group of stations can transmit data, while others are forbidden to access the channel. The RAW is supposed to be used to protect TWT transmission periods, which resembles the new Quieting Framework function to protect R-TWT transmission periods. However, the RAW does not control legacy devices. It only works in systems consisting of devices compliant with 802.11ah and cannot be used to protect R-TWT in heterogeneous Wi-Fi networks.

Unfortunately, many studies confirm that real devices do not fully correspond to the standard. For example, papers [22,23,24] show that real devices perform carrier sense differently from the standardized approach, and such deviation is widespread. In addition to the problem of inconsistency with the standard behavior, there are issues of coexistence between modern and legacy 802.11 devices. Papers [25,26,27,28] consider cases when legacy devices partially or completely disrupt the work of new Wi-Fi mechanisms. The studies show that such cases are widespread, and possible problems with similar behavior are not ignored.

This experimental study is the first to investigate the behavior of modern Wi-Fi devices that protect real-time transmissions, e.g., virtual reality traffic with R-TWT and multiple short Quiet Intervals. Specifically, we consider two conditions previously not found in the literature: the use of short Quiet Intervals and the simultaneous use of multiple Quiet Intervals. Our findings contribute to the understanding of how modern devices implement the Quieting Framework and shed light on the feasible effectiveness of the R-TWT mechanism in future Wi-Fi 7 networks.

## 3. Experiment

In the latest draft version D4.1 of the 802.11be amendment, the Quieting Framework is used to protect R-TWT SPs. To prevent SP occupation by other devices, protecting its beginning is sufficient. The draft specifies that the protection is achieved using a Quiet Interval with a duration of 1 TU, which is supported by legacy devices. Many emerging applications, e.g., virtual reality, generate quasi-periodic traffic and require frequent R-TWT SPs protected by Quiet Intervals. Note that the period of Quiet Intervals described in a single Quiet Element is a multiple of the Beacon Interval, i.e., at least hundreds of ms, which is much higher than the interval between two video frames in a virtual reality application, i.e., a dozen ms. Thus, a single Quiet Element per Beacon Interval is not sufficient, even if the corresponding Quiet Interval is very long, and such a flow requires multiple Quiet Elements.

In this study, we investigated the behavior of modern Wi-Fi devices in the scenarios relevant to R-TWT. Specifically, we studied whether the devices abide by (i) a single Quiet Interval of long duration, (ii) a single Quiet Interval with a duration of 1 TU, and (iii) multiple Quiet Intervals, which are identified as Tests 1–3. Test 3 consisted of two subtests: we independently investigated how devices execute multiple Quiet Intervals when they are scheduled for the same or different Beacon Intervals.

**Figure 2:**
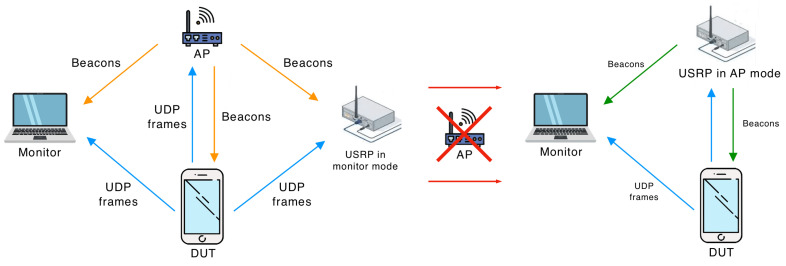
Two stages of experiment. In the first stage, the USRP listens to the AP beacons and stores them. In the second stage, the USRP sends beacons instead of the AP with added Quiet Element(s) with required parameters.

To perform the experiments, we designed a testbed, displayed in Figure 2. It consists of (i) an 802.11 device under testing (DUT); (ii) a monitor, which is a MacBook Pro with Wireshark [29] that captures and analyzes frames sent in the channel; and (iii) an AP. We used two devices for the AP: an Aruba IAP-207 and a Universal Software Radio Peripheral (USRP) [30] with a modified 802.11 Application Framework.

On the one hand, the Aruba IAP-207 can be configured to send beacons that make the Quieting Framework mandatory in the network. However, making it announce specific Quiet Elements is complicated. On the other hand, with USRP, we can send any frame, but the implementation of full authentication and association frameworks requires much effort. Thus, we implemented the idea from [31]. Specifically, in the beginning, the DUT associates with the Aruba IAP-207 access point, which sends beacons. The USRP works in the monitor mode: it receives the beacons and saves them. Then, the Aruba IAP-207 is switched off, and the USRP automatically starts sending beacons on behalf of the Aruba IAP-207. The content of beacons is the same except for the added Quiet Elements and recalculated frame check sequence.

Both the Aruba IAP-207 and USRP are configured to send beacons with a default period of 100 TUs. During the experiment, DUT was associated with the AP (Aruba IAP-207 or USRP) and transmitted saturated UDP traffic to it. UDP did not generate transport layer acknowledgments and thus does not produce unwanted downlink traffic. The UDP packet size is 1000 bytes. The AP only acknowledges data frames at the MAC layer and periodically sends beacons.

For each experiment, we recorded all frames sent in the channel and processed them as follows. First, we determined beacons with Quiet Elements where the Quiet Count equaled one because they set the Quiet Interval(s) in the following Beacon Interval (BI). Next, we determined the beacon inside this following BI where the Quiet Interval should occur and calculated its TBTT. Since the AP generates beacons at times k · BI, k = 0, 1, 2, ..., TBTT was determined as the timestamp value in the beacon rounded down to the nearest multiple of the BI. This TBTT determines the beginning of the Beacon Interval, where the Quiet Interval is performed. Then, we divided this beacon into 100 TUs starting from TBTT and calculated the number of frames sent by the device in each TU. We refer to this value as “frame intensity”. As a result, we obtained how the frame intensity value changes within the considered Beacon Interval. Finally, we averaged the obtained number of frames in a specific TU of a Beacon Interval over multiple BIs. For better statistical significance, every experiment had two runs, and we processed at least 100 BIs in every run. Averaging was performed over all obtained Beacon Intervals.

## 4. Numerical Results

With the designed testbed, we investigated how modern Wi-Fi devices support the Quieting Framework. Each DUT underwent three tests listed at the beginning of Section 3. Based on the obtained results and the vendors, we grouped all the considered devices into five groups; see Table 1. Let us consider the results in detail.

### 4.1. Apple Devices

**Figure 3:**
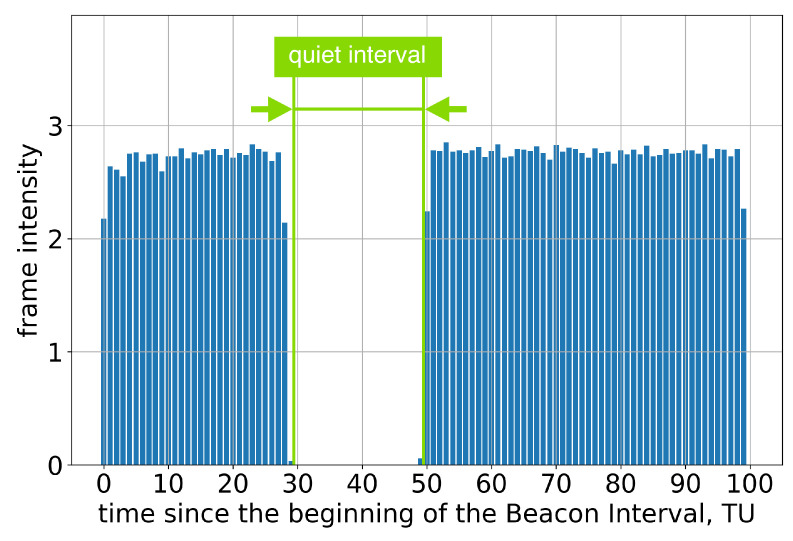
The average frame intensity of Group A devices in Test 1.

Group A consists of Apple devices, which run the macOS and iOS operating systems. These devices claim to support SM and are therefore expected to support the Quieting Framework in accordance with the standard. However, only Test 1 successfully passes. Specifically, if the AP advertises only one long Quiet Interval per two or more Beacon Intervals, the devices do not transmit during Quiet Intervals, as intended; see Figure 3.

**Figure 4:**
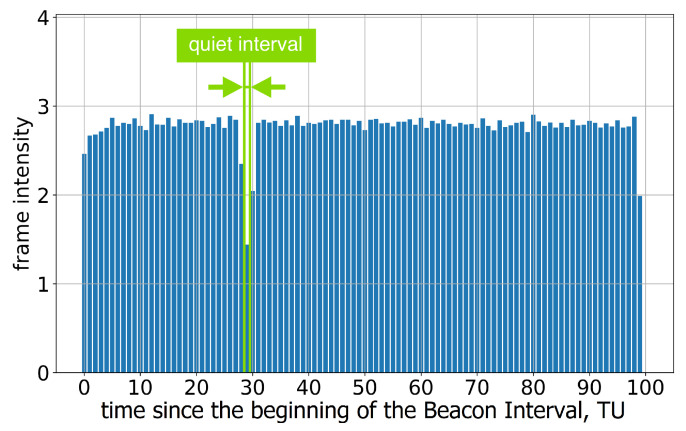
The average frame intensity of Group A devices in Test 2.

If the length of the Quiet Interval is 1 TU, the position of this interval within the Beacon Interval becomes too inaccurate; see Figure 4. This means that the stations assigned to the R-TWT may wait for the channel to become idle or even experience collisions. The problem becomes more significant in the case of several neighboring legacy stations with independent errors in the Quiet Interval position because the Quiet Intervals at these stations may not coincide.

**Figure 5:**
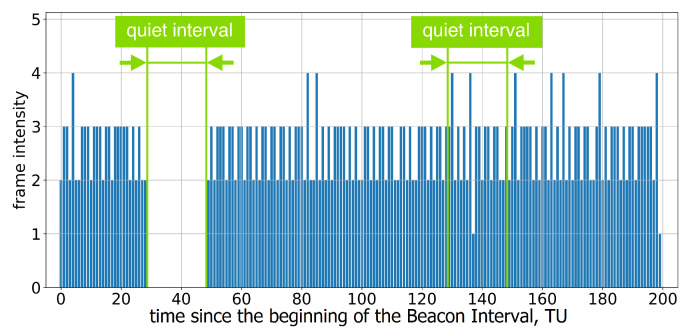
Example of frame intensity of Group A devices in Test 3 on two consecutive Beacon Intervals, each of which has an allocated Quiet Interval.

**Figure 6:**
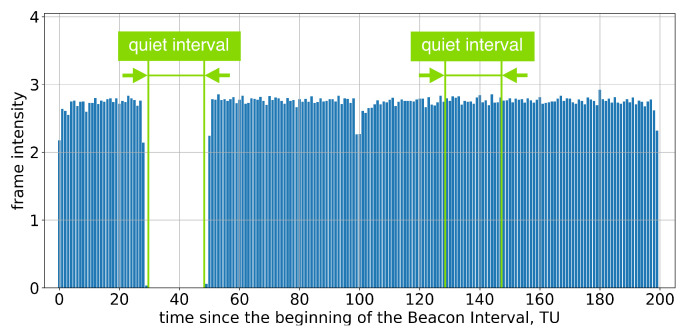
The average frame intensity of Group A devices in Test 3 on two consecutive Beacon Intervals, each of which has an allocated Quiet Interval.

If the AP advertises a single Quiet Interval in every BI, every second Quiet Interval is ignored; see Figure 5 and Figure 6.

**Figure 7:**
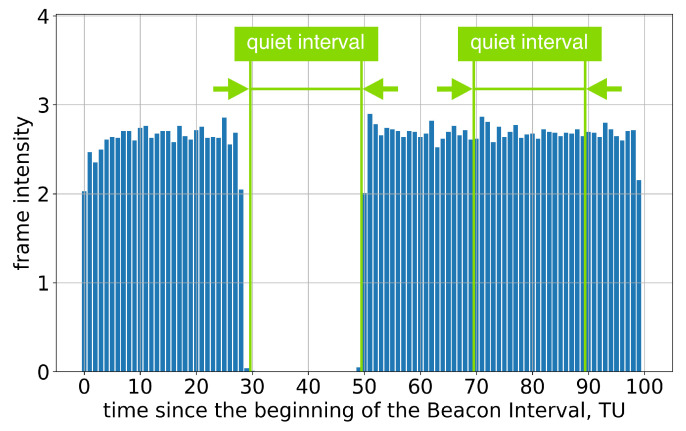
The average frame intensity of Group A devices in Test 3 with two Quiet Intervals in a Beacon Interval.

A similar problem occurs when the AP advertises more than one Quiet Interval in a BI. Devices remain silent only during the Quiet Interval, as instructed by the first Quiet Element in the beacons; see Figure 7. These findings indicate that the devices in Group A lack support for multiple Quiet Intervals, which is essential for R-TWT.

A possible reason for the observed behavior, which corresponds to the performed tests, is the oversimplified implementation of the Quieting Framework. Specifically, the devices store information of only one Quiet Interval and do not update it until the Quiet Interval comes. We assume that the hardware part of the chipset architecture has only one set of elements for Quieting purposes, such as memory slots and counters. So, the device reserves these hardware resources for one Quiet Element received and frees them when the respective Quiet Interval finishes. Until then, all other Quiet Elements are ignored due to resource occupancy.

### 4.2. Android Devices Based on SoC Qualcomm

Group B consists of several Android mobile devices based on the Qualcomm system-on-chip (SoC). Like the devices in Group A, they also indicate SM support but do not support the advertisement for multiple Quiet Intervals. In Test 1, we discovered that the Quiet Intervals are only executed correctly if their period is at least ten BIs. If the Quiet Interval is requested more often than once per ten BIs, the device may accidentally ignore some Quiet Intervals.

### 4.3. Android Devices without Quieting Framework Support

Group C consists of Android mobile devices that do not support the Quieting Framework entirely, although some of them may raise the SM flag. For example, during association, the Xiaomi Redmi Note 4 indicates that it does not support SM and the Quieting Framework. In contrast, the Huawei P40 claims to support SM but never remains silent in any tests. We assume that the absolute absence of Quiet Interval observance is connected with misconceptions in Spectrum Management implementation. The vendors might implement Spectrum Management partially, with the support for the transmit power control (TPC) mechanism but without the support for DFS. In such cases, devices do not have a radar detection framework or the Quieting Framework.

### 4.4. Acer Laptop with Qualcomm Atheros Wi-Fi Chip

**Figure 8:**
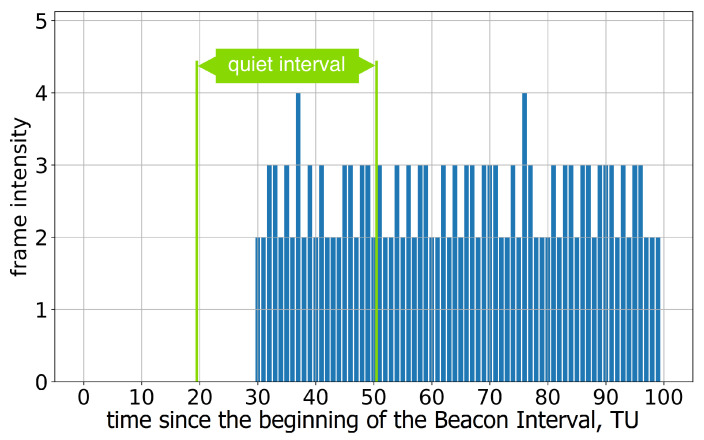
The average frame intensity of Group D devices in Test 1 with a single Quiet Interval.

Group D consists only of one device, the Acer Aspire 5, equipped with the Qualcomm Atheros Wi-Fi chip. Interestingly, the results for this device depend on the operating system (OS). In particular, the DUT running Windows does not declare that it supports SM and does not avoid transmissions during the Quiet Intervals in the tests. The device operated by Linux tries to authenticate to the AP with the raised SM flag. However, it incorrectly estimates the Quiet Interval position (see Figure 8), while the Quiet Interval duration is correct. Such behavior has been observed in several repeated experiments for different values of Quiet Element; the device changes the Quiet Interval duration in accordance with the Duration field but completely ignores the Offset field.

### 4.5. Laptops with Intel Wi-Fi Chips

Group E consists of laptops with Intel Wi-Fi chips. Similarly to Group D, the support for the SM flag depends on the operating system. However, unlike Group D, the devices do not support the Quieting Framework in any experimental scenarios and with any considered OS.

### 4.6. Experimental Results Overview

From the obtained experimental data, we conclude that the majority of tested devices do not support the Quieting Framework entirely. The other studied devices only partially support the Quieting Framework. Specifically, none of them support more than two Quiet Intervals per Beacon Interval. Some devices incorrectly estimate the position of the Quiet Interval within the Beacon Interval.

These findings raise questions about the use of the Quieting Framework jointly with R-TWT to protect transmissions of real-time flows. First, it seems impossible to protect the channel reservations from interference induced by legacy devices if channel reservations occur more often than once per two Beacon Intervals, which equals five reservations per second with default parameters. Such rare reservations are far from sufficient for many real-time streams. Second, many devices incorrectly locate the position of the Quiet Interval in time, which may even increase the interference during Quiet Intervals for the following reasons. Let their traffic be fixed. These devices avoid channel access during the wrong time intervals. Given a fixed traffic load, transmission probability is increased during the remaining time, including the real Quiet Intervals.

Summing up, the Quieting Framework cannot protect R-TWT transmissions from transmissions of legacy devices.

## 5. Conclusions

In the work, we have studied whether heterogeneous Wi-Fi 7 (802.11be) networks can rely on the Quieting Framework as a backward-compatible way to protect R-TWT real-time transmissions from interference induced by legacy devices. With a designed experimental setup, we show that the majority of the existing devices do not support the Quieting Framework with sufficient accuracy for R-TWT. Specifically, we present and classify a list of inconsistencies between the real operation of devices and the standard. Our results raise questions about the viability of this protection mechanism and, consequently, the effectiveness of the R-TWT mechanism.

We can identify several promising approaches as an alternative solution to the problem at hand. One of them is implementing different EDCA rules for modern and legacy devices, which can easily diminish the problem, but protection can still be compromised by random chance. Another direction is modifying self-CTS frames for channel reservation. Virtual reservation is a powerful backward-compatible tool, but it may cause high channel waste in many scenarios. Finally, R-TWT can be utilized in a dedicated channel without legacy devices, which can be achieved with a multi-link feature from 802.11be. However, this approach will necessitate additional hardware changes to the device, and the new channel will be underutilized most of the time. Nevertheless, the effectiveness of all approaches needs to be further researched and enhanced in future amendments. We hope to bring attention to this issue and encourage the community to work toward finding a solution.

## Figures and Tables

**Table 1:** Experimental results.

Group	Device	OS	Wi-Fi Chip	SM Flag	Tests		
					1	2	3
A	Apple MacBook Air 2012	macOS Catalina 10.15.7	BCM43xx 1.0(0x14E4, 0xE9)	●	●	A	-
	iPad Pro 2018	IOS 15.3.1	Murata/Apple 339S00551	●	●	A	-
	iPhone 12	IOS 15.3.1	USI 339S00761	●	●	A	-
B	Samsung Galaxy Note 10	Android 12	SoC Snapdragon 855	●	A	A	-
	OPPO Reno 5	Android 12	SoC Snapdragon 720G	●	A	A	-
	Xiaomi MI9T	Android 10	SoC Snapdragon 730	●	A	A	-
C	Xiaomi Redmi Note 4	Android 6.0.1	SoC Snapdragon 625	-	-	-	-
	Huawei P40	EMUI 12.0.0	Kirin W650	●	-	-	-
D	Acer Aspire 5	Windows 10 Pro 21H2	Qualcomm Atheros QCA61x4A	-	-	-	-
		Linux Mint 20.1		●	A	A	-
E	Lenovo ThinkPad P51	Windows 10 Pro 20H2	Intel Dual Band Wireless-AC 8265	-	-	-	-
		Linux 6.0.0-kali3		●	-	-	-
	Lenovo Yoga 730-13IWL	Windows 10 21H2	Intel Wireless-AC 9260	-	-	-	-
		Linux 6.0.0-kali3		●	-	-	-

●: Present/passed; A: Inaccurate position of the QI; –: Absent/did not pass.

## Abbreviations

The following abbreviations are used in this manuscript:

AP	Access Point
BI	Beacon Interval
DUT	Device Under Testing
EDCA	Enhanced Distributed Channel Access
R-TWT	Restricted Target Wake Time
SM	Spectrum Management
SP	Service Period
TBTT	Target Beacon Transmission Time
TU	Time Unit
